# ATG14‐Mediated SNARE Complex Activation Promotes ΔFosB Degradation to Ameliorate Levodopa‐Induced Dyskinesia

**DOI:** 10.1111/jnc.70431

**Published:** 2026-04-09

**Authors:** Yi Wu, Ke Liu, Zhaoyuan Zhang, Zhuoran Ma, Zhicheng Tang, An Chang, Haoxuan Ouyang, Heng Zhai, Xuebing Cao, Yan Xu

**Affiliations:** ^1^ Department of Neurology Union Hospital, Tongji Medical College, Huazhong University of Science and Technology Wuhan Hubei China; ^2^ Department of Neurology Jiangsu Province Hospital of Chinese Medicine, Affiliated Hospital of Nanjing University of Chinese Medicine Nanjing China; ^3^ Department of Neurology the First Medical Center, Chinese PLA General Hospital Beijing China

**Keywords:** ΔFosB, ATG14, autophagy, levodopa‐induced dyskinesia, SNARE

## Abstract

The chronic accumulation of ΔFosB in striatal medium spiny neurons has been implicated as a pivotal contributor to the pathogenesis of levodopa‐induced dyskinesia (LID). While recent studies have implicated autophagy in the degradation of ΔFosB and the amelioration of LID, the precise mechanisms remain elusive. We induced LID in a unilateral 6‐hydroxydopamine‐lesioned parkinsonism rat model via chronic levodopa treatment. To modulate the autophagy pathway, we overexpressed ATG14 in the striatum of LID rats and administered chloroquine, an autophagy inhibitor, peripherally. We assessed LID severity using abnormal involuntary movements (AIMs) scores. Western blotting, real‐time quantitative polymerase chain reaction, immunofluorescence, immunohistochemistry, transmission electron microscopy, and Golgi staining were employed to measure autophagy flux, synaptic alterations, and ΔFosB levels. Chronic levodopa treatment reduced ATG14 and SNARE complex (STX17, SNAP29, and VAMP8) levels, disrupted their interaction, impaired autophagy flux, affected synaptic function, and led to ΔFosB accumulation in the striatum of PD rats. Upregulating ATG14 in the striatum of LID rats improved AIMs scores, facilitated SNARE‐mediated autophagosome‐lysosome fusion, restored synaptic deficits, and promoted ΔFosB degradation. However, these beneficial effects of ATG14 upregulation were negated by chloroquine administration. Our findings suggest that upregulating ATG14 enhances SNARE formation, promoting autophagy flux and thereby reducing LID occurrence by facilitating ΔFosB degradation.

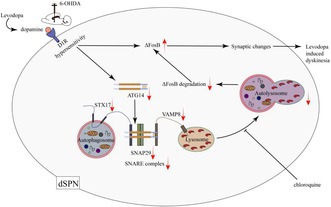

## Graphical abstract

Understanding the mechanisms underlying ΔFosB accumulation in direct‐pathway striatal projection neurons (dSPNs) is crucial for elucidating levodopa‐induced dyskinesia (LID). Our study reveals that ATG14 upregulation and the concomitant SNARE complex activation promote ΔFosB degradation. This finding potentially paves the way for innovative therapeutic strategies targeting ΔFosB in LID.

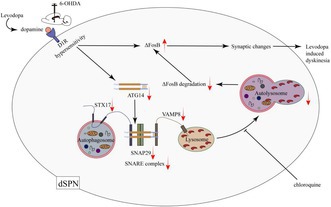

**Abbreviations**
6‐OHDA: 6‐hydroxydopamine
AAV: adeno‐associated virus
AIMs: abnormal involuntary movements
ALO: axial limb and orolingual
ATG14: autophagy‐related 14
BSA: bovine serum albumin
CQ: chloroquine
D1R: dopamine D1 receptors
dSPNs: direct‐pathway striatal projection neurons
GluR1: alpha‐amino‐3‐hydroxy‐5‐methyl‐4‐soxazole propionic acid receptor subtype 1
IF: immunofluorescence
IHC: immunohistochemistry
L‐DOPA: levodopa
LID: levodopa‐induced dyskinesia
MFB: medial forebrain bundle
PD: Parkinson's disease
PSD95: postsynaptic density 95
RRID: research resource identifier
RT‐qPCR: real‐time quantitative polymerase chain reaction
SAP97: synapse‐associated protein 97
SNAP29: SNARE‐binding protein synaptosomal‐associated protein of 29 kDa
SNARE: soluble N‐ethylmaleimide‐sensitive factor attachment protein receptor
STX17: syntaxin 17
TEM: transmission electron microscopy
VAMP8: vesicle associated membrane protein 8
WB: western blotting

## 1 Background

Parkinson's disease (PD) ranks as the second most prevalent neurological disorder, neuropathologically distinguished by the degeneration of dopaminergic neurons in the substantia nigra. Thus far, there have been virtually no interventions capable of halting or reversing its progression. Levodopa (L‐DOPA) therapy has long been a cornerstone in the management of PD. However, levodopa‐induced dyskinesia (LID) develops in over 50% of PD patients following 5 years of L‐DOPA treatment (Bandopadhyay et al. 2022). A primary factor contributing to the onset of LID is the sensitized dopamine D1 receptors (D1R) located on direct‐pathway striatal projection neurons (dSPNs) (Falkenburger et al. 2022).

ΔFosB plays a pivotal role in LID. Studies conducted on both LID patients and animal models have revealed a marked increase in ΔFosB expression within the striatum, and this elevation directly correlates with the progression of LID severity (Zamanian et al. 2025). Upregulating ΔFosB in the striatum leads to the rapid onset of LID and associated unstable SPNs firing upon exposure to L‐DOPA (Cao et al. 2010; Beck et al. 2019). Conversely, the inactivation of ΔFosB in the striatum reverses LID without compromising the antiparkinsonian effects of L‐DOPA (Beck et al. 2021; Engeln et al. 2016; Berton et al. 2009). The lack of degron domains shields ΔFosB from ubiquitination degradation (Robison and Nestler 2022). Nonetheless, ΔFosB levels are notably elevated only during morbid conditions, remaining low in normal states (Robison and Nestler 2022), suggesting additional degradation pathways for ΔFosB.

Autophagy, a process that digests abnormal cytosolic components, efficiently removes insoluble and aggregated proteins (Vargas et al. 2023). Recent findings suggest that D1R hyperactivation increases the autophagy‐specific substrate p62 in LID (Feyder et al. 2021), indicating autophagy impairment in the striatum of LID. Our research further demonstrates that activating autophagy facilitates ΔFosB degradation, improves abnormal neuronal firing, and reduces LID severity (Liu et al. 2024). Conversely, inhibiting autophagy with chloroquine (CQ) exacerbates ΔFosB accumulation and reverses the aforementioned improvements in neuronal firing and dyskinogenic behavior (Liu et al. 2024). CQ disrupts autophagic flux by impeding autophagosome‐lysosome fusion (Mauthe et al. 2018). Additionally, autophagy‐related 14 (ATG14), also known as Beclin 1‐associated autophagy‐related key regulator, plays a critical role in autophagy (Xiong et al. 2012). Previous studies have revealed that ATG14 directly interacts with the soluble N‐ethylmaleimide‐sensitive factor attachment protein receptor (SNARE) complex, consisting of syntaxin 17 (STX17), SNARE‐binding protein synaptosomal‐associated protein of 29 kDa (SNAP29), and vesicle associated membrane protein 8 (VAMP8), to regulate autophagosome‐lysosome fusion (Liu et al. 2015).

The specific mechanism of autophagy in ΔFosB degradation remains unclear in LID. In the present study, we sought to explore the function of ATG14 in LID and further enhance the ATG14‐mediated SNARE pathway through transgenic modulation to ascertain whether activating ATG14 can facilitate the elimination of ΔFosB and consequently mitigate LID.

## 2 Methods

### 2.1 Animals

Adult male Sprague–Dawley rats (8–9 weeks old, weight, 200–220 g) were purchased from Liaoning Changsheng Biotechnology Co. Ltd. (Liaoning, China). All rats were maintained in a specific‐pathogen‐free house under a constant humidity (60% ± 5%) and temperature (22°C ± 1°C) with adequate food and water. The rats were kept in polypropylene cages (40 cm × 30 cm × 20 cm), with 3 rats per cage. The experiment was authorized by the Experimental Animal Management Committee of Tongji Medical College of Huazhong University of Science and Technology (ethical approval reference number: 3865). No sample size calculation was performed. According to the resource equation method, the appropriate sample size was determined by ensuring the degrees of freedom E (total animals − total groups) ranged from 10 to 20 (Charan and Kantharia 2013). In this study, 13 or 16 animals per group were assigned to various assays, with 3 biological replicates for molecular assays except co‐Immunoprecipitation (Co‐IP, *n* = 1). The calculated E values of 10 and 14 were within the acceptable range.

### 2.2 Stereotaxic Lesions and Apomorphine Test

The rats were deeply anesthetized with 2% sodium pentobarbital (50 mg/kg) and then gently placed on a stereotaxic apparatus. 6‐hydroxydopamine (6‐OHDA, Cat. No. H4381, Sigma‐Aldrich, 2 μg/μL) and apomorphine (Cat. No. PHR2621, Sigma‐Aldrich, 0.05 mg/kg, 0.1 mg/mL, s.c.) were dissolved in sterile saline supplemented with 0.02% ascorbic acid. Except for the control group, all rats underwent slow lesioning with a total of 4 μL of 6‐OHDA delivered to two points within the right medial forebrain bundle (MFB) via a 10 μL microsyringe at a rate of 1 μL/min. Rats in the sham group received an equivalent volume of sterile saline containing 0.02% ascorbic acid into the MFB. The specific coordinates used were: AP, −4.40 mm and ML, −1.5 mm from the bregma; DV, 7.85 ± 0.05 mm below the dura. A heating pad maintained the animals' body temperature until they awoke.

Following a 2‐week recovery period, contralateral rotations were induced in rats following subcutaneous injection of apomorphine, and the rotational behavior was continuously observed for 30 min. Rats that exhibited ≥ 7 turns per minute in response to apomorphine were considered to have established PD. For the remainder of this paper, PD group refers to animals with dopaminergic neuron depletion that have not received L‐DOPA treatment. The construction of the adeno‐associated virus (AAV) was commissioned to Shanghai Heyuan Biotechnology Co. Ltd. For the transgenic rats, additional striatal injections of pAAV‐CMV‐EGFP‐P2A‐ATG14‐3×FLAG‐WPRE (abbreviated as AAV‐ATG14; titer: 1.62E+12 v.g./mL) and pAAV‐CMV‐EGFP‐P2A‐3×FLAG‐WPRE (abbreviated as AAV‐GFP; titer: 2.82E+13 v.g./mL) were administered after the apomorphine test at the following coordinates: AP, +1.2 mm and ML, −2.4 mm from the bregma; DV, 4 mm below the dura. Each transgenic rat received 4 μL of AAV into the right striatum using a microsyringe at a rate of 1 μL/min. After stereotaxic injection, animals were monitored for vital signs such as breathing rate and reflexes during recovery to detect any signs of pain or distress; meanwhile, a heating pad was used to maintain body temperature, and 2% lidocaine ointment was applied once postoperatively to provide analgesia. No rats were excluded from the study due to pain or distress behaviors; 6 rats were excluded based on the rotation test, and none died during the experiments. Since extra rats were prepared according to the previous modeling success rate, no additional replacement experiments were required. No randomization was performed to allocate subjects in the study.

### 2.3 Drug Treatments

L‐DOPA (Cat. No. D1507, Sigma‐Aldrich, 12 mg/kg, 12 mg/mL, i.p.), benserazide (Cat. No. B7283, Sigma‐Aldrich, 6 mg/kg, 6 mg/mL, i.p.), and CQ (Cat. No. C6628, Sigma‐Aldrich, 60 mg/kg, 60 mg/mL, i.p.) were also dissolved in sterile saline before use.

In order to induce dyskinesia, rats were challenged intraperitoneally with L‐DOPA and benserazide dissolved in saline for 14 consecutive days. For the LID group, L‐DOPA and benserazide began on the second day after the apomorphine test. In the transgenic groups, the same L‐DOPA and benserazide treatment commenced 4 weeks after virus injection. CQ was further administered in rAAV‐ATG14 rats after 2‐week L‐DOPA and benserazide treatment. An equal volume of solvent was given for all control groups. The experimental design, as outlined in Figure 3A, provides a visual representation of this protocol. A total of 93 rats were included in the study, with an initial number of 13 rats in each of the Sham, PD and LID groups, and 16 rats in each of the AAV‐GFP, AAV‐ATG14 and AAV‐ATG14+CQ groups. No blinding was performed.

Measures were taken to prevent scar formation. In brain surgery, microsyringes were used to slowly inject 6‐OHDA and AAV (with the injection volume controlled at 1.5–2 μL per site); the needle was left in place for 5 min and withdrawn slowly after injection. For 2‐week intraperitoneal injections, sites were rotated among the four abdominal quadrants.

### 2.4 Cylinder Test

The cylinder test was conducted prior to and post 6‐OHDA lesion to evaluate the motor function of rats. Briefly, these rats were gently placed into tailored cylinders (24 cm in diameter and 16.5 cm in height). We documented every touch made by their unilateral and bilateral forelimbs on the cylinder walls until a total of 20 contacts were registered or until 10 min had elapsed. The formula used for calculation was: [(1/2 bilateral + left)/(left + right + bilateral)] × 100%.

### 2.5 Abnormal Involuntary Movements Assessment (AIMs)

AIMs scores were recorded every other day since L‐DOPA treatment as described previously. AIMs were scored every 20 min over a 140‐min period following the administration of L‐DOPA. The total AIM scores are composed of axial, limb and orolingual AIMs (namely ALO AIMs). Their severity was defined as follows: 0, absence of AIMs; 1, occasional, duration < 30 s; 2, frequent, duration > 30 s; 3, continuous but stoppable by external stimuli; 4, continuous and unstoppable via external stimuli.

### 2.6 Protein Extraction

Right dorsal striata were isolated by gross anatomical dissection, with approximate coordinates: AP, −1.4 to 2.5 mm and ML, −2.2 to −3.8 mm from the bregma; DV, −3.2 to −4.4 mm below the dura. These tissues were then mechanically ground into homogenate in lysis buffer containing PMSF, phosphatase inhibitor A, phosphatase inhibitor B, and protease inhibitor cocktail at a volume ratio of 100:1:1:1. After sonication and centrifugation, the supernatant was collected as the total protein extract and then denatured via a metal bath. The protein concentration of the supernatant was determined by the bicinchoninic acid kit (Cat. No. AR1189, Boster).

To separate out the plasma membrane, we used the Membrane and Cytosol Protein Extraction kit (Cat. No. P0033, Beyotime). As per the manufacturer's instructions, rat right striatal tissue was first homogenized in solution A, which had been pre‐supplemented with PMSF, and then centrifuged at 700 × g for 12 min at 4°C. The supernatant was subsequently collected and centrifuged again at 14000 × g for 35 min at 4°C. The resulting supernatant, identified as the cytoplasm fraction, was removed. The deposit was fully re‐suspended in solution B and centrifuged once again. The supernatant from this step, designated as the plasma fraction, was collected and then denatured.

### 2.7 Western Blotting (WB)

Protein samples were detected via electrophoresis and then transferred to PVDF membranes, with proteins < 20 kDa separated on 12.5% separating gels (Cat. No.: PG113, Yamay) and all other proteins on 10% separating gels (Cat. No. PG112, Yamay). After blockage with 5% bovine serum albumin (BSA) in TBST for 1 h at room temperature, the membrane underwent incubation with primary antibodies overnight at 4°C with gentle shaking, and then with secondary antibody for 1 h at room temperature with gentle shaking. Between each incubation step, the membranes were washed three times for 10 min each with TBST. Chemiluminescence signals were detected via the horseradish peroxidase‐mediated chemiluminescence method (ClinX, China), while ImageJ software was utilized for analyzing the corresponding results. Antibodies involved in this study are listed in Table S1. Original WB images are presented in Figures S1 and S2.

### 2.8 Co‐IP

In brief, right striatal tissue was isolated and mechanically ground in lysis buffer for immunoprecipitation. After centrifugation, the supernatant was incubated with the primary antibody ATG14 (2 μg per 2000 μg total protein; Cat. No. 19491‐1‐AP, Proteintech) overnight at 4°C. Next, Protein A+G Agarose (30 μL per 100 μL the supernatant; Cat. No. P2012, Beyotime) was added to the sample and incubated for an additional 4–6 h before denaturation for further western blotting.

### 2.9 Real‐Time Quantitative Polymerase Chain Reaction (RT‐qPCR)

Following the manufacturer's guidelines, RNA Isolation Kit (Cat. No. RC112, Vazyme) and Reverse Transcription Kit (Cat. No. R323, Vazyme) were used to extract cDNA. Afterward, we prepared reaction mixtures by combining cDNA, primers, ddH_2_O, Dye 1, and ChamQ SYBR qPCR Master Mix (Cat. No. Q311, Vazyme), and then dispensed the mixtures into PCR plates. We conducted RT‐qPCR and acquired the respective CT values via the StepOnePlus RT‐qPCR system. The primers for rat ΔFosB were F: 5′‐GGCCTAGAAGACCCCGAGAA‐3′ and R: 5′‐TCCTCTTCGAGCTGATCCGT‐3′. The primers for rat GAPDH were F: 5′‐GCAAGTTCAACGGCACAG‐3′ and R: 5′‐GCCAGTAGACTCCACGACAT‐3′.

### 2.10 Tissue Preparation and Immunohistochemistry (IHC)

Briefly, on the last day of experiment, the rats were anesthetized and then perfused with saline and 4% paraformaldehyde. The brains were subsequently acquired, paraffin‐embedded, and sectioned into 4–5 μm thick slices. Sections were collected from −1.4 to +2.5 mm relative to bregma. The largest coronal slices at the rostral level were selected for the subsequent step. The brain sections sequentially underwent xylene deparaffinization, gradient alcohol rehydration, antigen retrieval using a citrate buffer, quenching of endogenous peroxidase with 3% hydrogen peroxide and blocking with 5% BSA for 1 h at room temperature. Then slides were incubated with diluted primary antibodies at 4°C overnight. After washing, the appropriate secondary antibodies were used to incubate above sections at room temperature for 1.5 h. After washing again, 3,3‐diaminobenzidine solution and hematoxylin were applied to stain and counterstain tissue slides respectively. Subsequently, the tissue slides underwent gradient alcohol dehydration and xylene transparency processes. Ultimately, they were sealed with neutral resin and then examined under a microscope. The results from the right dorsal striatum were analyzed with ImageJ software. For cytoplasmic‐localized molecules, the images were first converted to grayscale, followed by calibration of the optical density using the Uncalibrated OD function under the Calibrate tool. After threshold optimization, the Analyze function was applied to calculate the integrated optical density (IOD). For molecules expressed in the cell nucleus, images were first processed via color deconvolution for signal separation, followed by threshold adjustment to define positive staining. The Analyze Particles function was employed to quantify the number of positive cells and the total number of cells; the positive cell ratio was then calculated and utilized for further statistical analysis.

### 2.11 Immunofluorescence (IF)

Similar to the procedure for immunohistochemistry, the 4–5 μm thick slices underwent dewaxing, rehydration, antigen retrieval, and serum blockage before being incubated overnight at 4°C with primary antibodies. Next, the brain slices were rinsed and incubated at room temperature for 1.5 h. Upon further rinsing, the slices were sealed with an anti‐fluorescence quencher containing DAPI. The striatum images were scanned for further analysis. For monochromatic red or green images, the integrated density was measured by adjusting the threshold subsequent to image type conversion. For colocalization analysis, red and green images were merged using the Image Calculator function to generate a 32‐bit result merged image; the Lookup Tables function was then used to select the yellow color, and the integrated density was obtained via the Measure function. The mean value of the integrated density in the control group was calculated, and the integrated density of all samples was normalized against this mean value; the normalized data were used for statistical analysis.

### 2.12 Transmission Electron Microscopy (TEM)

TEM was employed to capture autophagic vacuoles and autolysosomes in the striatum of rats from each group. In the simplest terms, right striatal tissue was carefully dissected and immediately fixed in a 2.5% glutaraldehyde solution upon sacrificing the rats. Following this, the tissue was further fixed in 1% osmium tetroxide. It was then dehydrated using a graded ethanol series and embedded in epoxy resin, allowing for ultrathin sectioning at 50 nm. The tissue slices were subsequently stained by sequential immersion in 2% uranyl acetate saturated alcohol solution and 2.6% lead citrate solution. Finally, the prepared tissue sections were imaged using a transmission electron microscope (HITACHI, Japan). Autolysosomes and autophagic vacuoles in each sample were counted separately, and their respective proportions relative to the total number were calculated, normalized, and then subjected to statistical analysis. The same approach was adopted for the quantification of perforated and non‐perforated synapses.

### 2.13 Golgi Staining

We used the FD Rapid Golgi Stain Kit (Cat. No. PK401, FD Neuro Technologies) to stain dendritic spines. Following the removal of the striatum, it was incubated in a 1:1 mixture of solutions A and B for 3–4 weeks. Subsequently, the sample underwent further incubation in solution C for an additional 3–7 days. The tissue was then placed on an oscillating tissue slicer and cut into 100 μm thick sections. After drying in a dark environment, the slices were transferred to a staining solution consisting of a 1:1:2 mixture of solutions D, E, and double distilled water. Images were then captured using an optical microscope (Nikon, Japan). Perforated and non‐perforated synapses were quantified separately for each sample; their proportions relative to the total synapse count were calculated, normalized, and subsequently subjected to statistical analysis.

### 2.14 Statistical Analysis

All data were presented as mean ± SD and analyzed with GraphPad Prism 8 software. The D'Agostino‐Pearson test was used to confirm the normality of the data distribution, while the Brown‐Forsythe test and Bartlett test verified the homogeneity of variance. Comparisons between multiple groups were conducted using one‐way ANOVA, followed by Tukey's multiple comparisons test. To assess differences in behavioral scores over time, both between two groups and among multiple groups, two‐way ANOVA was employed, with Bonferroni's post hoc test. No test for outliers was conducted. Statistical significance was set at *p* < 0.05.

## 3 Results

### 3.1 Chronic L‐DOPA Treatment Suppressed ATG14 Pathway and Triggered Autophagy Dysfunction as Well as ΔFosB Accumulation in the Striatum of Parkinsonian Rats

**FIGURE 1:**
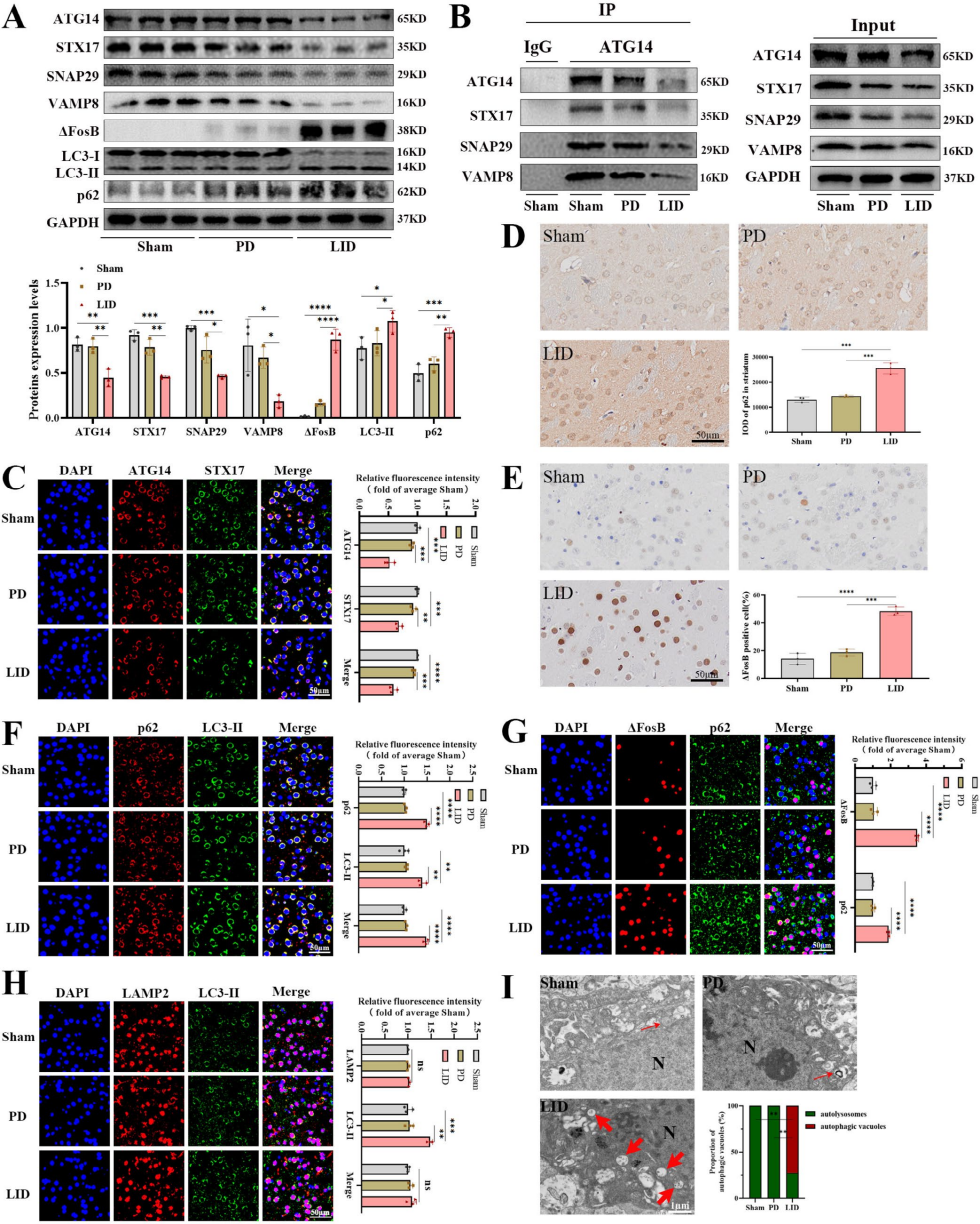
Long‐term levodopa treatment suppresses ATG14 (autophagy‐related 14) pathway, impairs autophagy, and promotes ΔFosB accumulation in LID (levodopa‐induced dyskinesia) rats. (A) The protein levels of ATG14, STX17 (syntaxin 17), SNAP29 (SNARE‐binding protein synaptosomal‐associated protein of 29 kDa), VAMP8 (vesicle associated membrane protein 8; SNARE), LC3, p62, and ΔFosB were detected by western blotting in the striatum of Sham, PD (Parkinson's disease), and LID group rats (*n* = 3). GAPDH as an internal reference. (B) The interaction levels of ATG14 and SNARE (soluble N‐ethylmaleimide‐sensitive factor attachment protein receptor) complex (namely STX17, SNAP29, and VAMP8) were detected by co‐immunoprecipitation in the striatum of each group (*n* = 1). (C, F, G, H) Representative immunofluorescence images of ATG14 and STX17 co‐localization, p62 and LC3‐II co‐localization, LAMP2 and LC3‐II co‐localization as well as p62 and ΔFosB co‐localization in the striatum of each group (*n* = 3; bar = 50 μm). (D, E) Representative immunohistochemical images of p62 and ΔFosB in the striatum of each group (*n* = 3; bar = 50 μm). (I) The representative transmission electron microscopy images of autophagic vacuoles and autolysosomes in each group (*n* = 3; N, nucleus; thin red arrows, autolysosomes; bold red arrows, autophagic vacuoles). (Bars represent the mean, error bars represent the SEM; One‐way ANOVA followed by Tukey multiple‐comparisons tests; *p < 0.05, **p < 0.01, ***p < 0.001, ****p < 0.0001; ns, no significant).

Marked dopaminergic denervation was observed in the right striatum of the PD and LID groups, confirming successful modeling (Figure S3). We analyzed p62 expression in the right striatum of each group, as p62 is a well‐known autophagic substrate. As shown in Figure 1A, immunoblotting results depicted augmented p62 protein abundance in LID rats compared to PD and Sham rats (*F*_(2,6)_ = 28.55, *p* = 0.0009). Immunohistochemistry and immunofluorescence also revealed a significant rise in p62 immune‐reactive cells in the LID group compared to Sham and PD group littermates, as depicted in Figure 1D (*F*_(2,6)_ = 70.68, *p* < 0.0001) and Figure 1F (*F*_(2,6)_ = 160.3, *p* < 0.0001), respectively. We further investigated LC3‐II expression, another established autophagy marker. Elevated LC3‐II protein expression was observed in the LID group compared to PD and Sham groups (Figure 1A; *F*_(2,6)_ = 7.552, *p* = 0.023). A concordant increase in LC3‐II immune‐reactive cells, along with p62, was found in the LID group than PD and Sham ones (Figure 1F; *F*_(2,6)_ = 21.24, *p* = 0.0019). TEM revealed that L‐DOPA treatment induced an increase in autophagic vacuoles (bold red arrows) and a decrease in autolysosomes (thin red arrows) within the striatum of LID rats (Figure 1I; *F*_(2,6)_ = 24.11, *p* = 0.0014) than control groups. These findings suggest reduced autophagy in LID. Moreover, the transition from autolysosomes to autophagic vacuoles indicates a potential failure in autophagosome‐lysosomal fusion.

We continually investigated specific mechanisms underlying the autophagosome‐lysosomal fusion within the right striatum of LID rats. Immunofluorescence analysis of Figure 1H revealed an increase in LC3‐II (labels for autophagosomes or autolysosomes; *F*_(2,6)_ = 31.74, *p* = 0.0006), and unaltered expression of LAMP2 (labels for lysosomes, late endosomes as well as autolysosomes; *F*_(2,6)_ = 3.504, *p* = 0.0981) and the co‐localization of LC3‐II with LAMP2 (*F*_(2,6)_ = 1.342, *p* = 0.3297) within the striatum of LID rats. This suggests that there was no significant change in lysosome number in these rats. Protein levels of ATG14 (*F*_(2,6)_ = 17.7, *p* = 0.003), STX17 (*F*_(2,6)_ = 43.83, *p* = 0.0003), SNAP29 (*F*_(2,6)_ = 28.41, *p* = 0.0009), and VAMP8 (*F*_(2,6)_ = 9.241, *p* = 0.0147) were remarkably reduced in the striatum of LID rats compared to PD and Sham counterparts (Figure 1A). Concordant with western blotting results, L‐DOPA treatment diminished immunoreactive ATG14 (*F*_(2,6)_ = 57.64, *p* = 0.0001), STX17 (*F*_(2,6)_ = 39.32, *p* = 0.0004) and co‐localization of ATG14 with STX17 (*F*_(2,6)_ = 86.2, *p* < 0.0001) in the striatum of LID rats (Figure 1C). As shown in Figure 1B and Figure S4, subsequent co‐immunoprecipitation findings further supported weaker interactions between ATG14 and SNARE complex (namely STX17, SNAP29, and VAMP8) in the striatum of LID rats. The observed halt in autophagosome‐lysosome fusion strongly correlates with ATG14‐mediated SNARE formation in the striatum of LID rats.

As evident in Figure 1A, immunoblotting displayed elevated ΔFosB protein levels in the right striatum of LID rats (*F*_(2,6)_ = 127.6, *p* < 0.0001). Parallels to the western blotting findings, immunohistochemistry unveiled a notably higher percentage of ΔFosB‐positive cells (Figure 1E; *F*_(2,6)_ = 95.54, *p* < 0.0001). Additionally, immunofluorescence analysis, presented in Figure 1G, exhibited a concurrent surge in both ΔFosB (*F*_(2,6)_ = 236.1, *p* < 0.0001) and p62 levels (*F*_(2,6)_ = 142.8, *p* < 0.0001), implying that autophagic deficits might lead to ΔFosB accumulation.

### 3.2 Chronic L‐DOPA Treatment Induced Synaptic Disorder in the Striatum of Parkinsonian Rats

**FIGURE 2:**
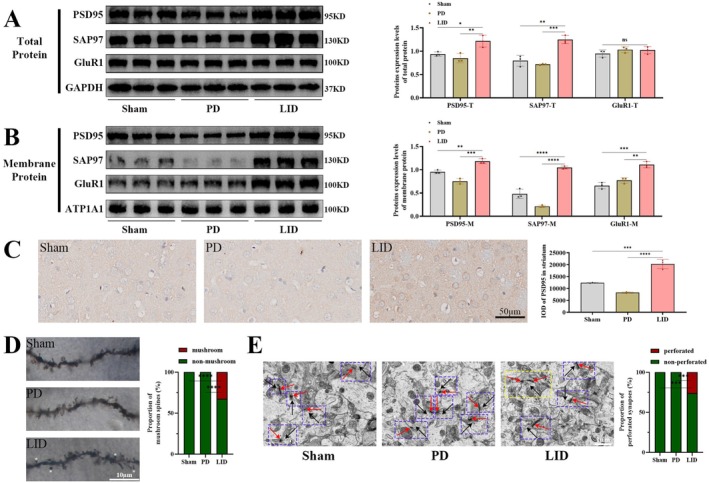
Long‐term levodopa treatment aggravated maladaptive synaptic structure in LID rats. (A) The total protein levels of PSD95 (postsynaptic density 95), SAP97 (synapse‐associated protein 97), and GluR1 (alpha‐amino‐3‐hydroxy‐5‐methyl‐4‐soxazole propionic acid receptor subtype 1) were detected by western blotting in the striatum of Sham, PD, and LID group rats (*n* = 3). GAPDH was used as an internal reference. (B) The membrane protein levels of PSD95, SAP97, and GluR1 were detected by western blotting in the striatum of each group (*n* = 3). ATP1A1 was used as an internal reference. (C) The representative immunohistochemical images of PSD95 in the striatum of each group (*n* = 3; bar = 50 μm). (D) The representative Golgi‐stained images of mushroom and non‐mushroom spines in each group (*n* = 3; bar = 10 μm; asterisk, mushroom spines). (E) The representative transmission electron microscopy images of perforated and non‐perforated synapses in each group (*n* = 3; bar = 1 μm; black arrows, presynaptic vesicles; red arrows, postsynaptic dense; yellow dashed box, perforated synapses; blue dashed box, non‐perforated synapses). (Bars represent the mean, error bars represent the SEM; One‐way ANOVA followed by Tukey multiple‐comparisons tests; *p < 0.05, **p < 0.01, ***p < 0.001, ****p < 0.0001; ns, no significant).

We analyzed maladaptive synapse, an established pathophysiology of LID. As shown in Figure 2A, total protein levels of postsynaptic density 95 (PSD95) (*F*_(2,6)_ = 11.64, *p* = 0.0086) and synapse‐associated protein 97 (SAP97) (*F*_(2,6)_ = 35.06, *p* = 0.0005) were increased in the striatum of LID rats relative to the other two groups, while the total protein of alpha‐amino‐3‐hydroxy‐5‐methyl‐4‐soxazole propionic acid receptor subtype 1 (GluR1) remained unchanged (*F*_(2,6)_ = 1.167, *p* = 0.3732). As for membrane protein levels shown in Figure 2B, both PSD95 (*F*_(2,6)_ = 54.06, *p* = 0.0001) and SAP97 (*F*_(2,6)_ = 142.1, *p* < 0.0001) increased further, and GluR1 (*F*_(2,6)_ = 37.51, *p* = 0.0004) also became enriched on the plasma membrane in the striatum of LID rats. As shown in Figure 2C, immunohistochemistry also revealed higher immunoreactive PSD95 in the striatum of LID rats (*F*_(2,6)_ = 85.75, *p* < 0.0001). Golgi‐stained striatum exhibited that the ratio of mushroom spines to non‐mushroom spines increased in the LID rats (Figure 2D; *F*_(2,6)_ = 240.1, *p* < 0.0001). TEM revealed that the ratio of perforated to non‐perforated synapses increased in LID groups (Figure 2E; *F*_(2,6)_ = 47.16, *p* = 0.002). The above results showed maladaptive synaptic structure accompanying the development of LID.

### 3.3 Striatal ATG14 Over‐Expression Alleviated AIMs, Whereas CQ Administration Hindered This Ameliorative Effect in LID Rats

**FIGURE 3:**
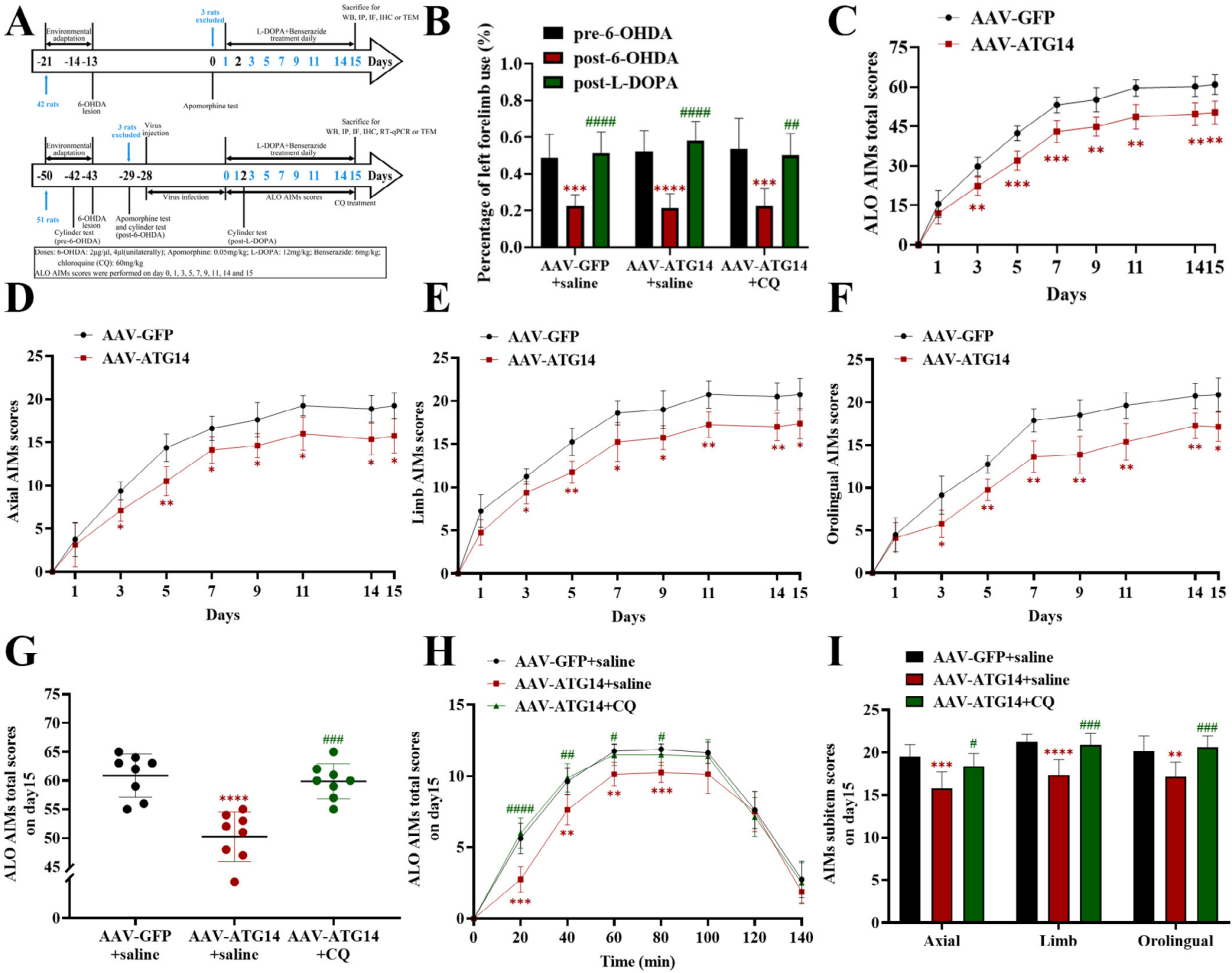
CQ (chloroquine) could worsen ALO (axial, limb, and orolingual) AIMs (abnormal involuntary movements) improved by ATG14 overexpression in LID rats. (A) The schematic timeline for 6‐OHDA (6‐hydroxydopamine) and ATG14 lesioning, CQ intervention, and ALO AIM scores assessment. (B) Left forelimb use in the cylinder test before (pre‐6‐OHDA) and after 6‐OHDA (post‐6‐OHDA) lesions and 30 min after the L‐DOPA (levodopa) intervention (post‐L‐DOPA) in AAV (adeno‐associated virus) ‐GFP+saline, AAV‐ATG14+saline, and AAV‐ATG14+CQ group rats (*n* = 8). (C) The line graphs of ALO AIMs total scores over time in AAV‐GFP and AAV‐ATG14 group (*n* = 8). (D) The line graphs of axial AIMs scores over time in each group (*n* = 8). (E) The line graphs of limb AIMs scores over time in each group (*n* = 8). (F) The line graphs of orolingual AIMs scores over time in each group (*n* = 8). (G) ALO AIM total scores for each group on day 15 (*n* = 8). (H) Time‐dependent ALO AIMs scores for each group on day 15 (*n* = 8). (I) Axial, limb, and orolingual AIMs scores for each group on day 15 (*n* = 8). (Bars represent the mean, error bars represent the SEM; two‐way repeated measures ANOVA with Bonferroni post hoc tests, one‐way ANOVA followed by Tukey multiple‐comparisons tests; *p < 0.05, **p < 0.01, ***p < 0.001, ****p < 0.0001 versus AAV‐GFP+saline group; ^#^ *p* < 0.05, ^##^ *p* < 0.01, ^###^ *p* < 0.001, ^####^ *p* < 0.0001 versus AAV‐ATG14+CQ group; ns, no significant).

To verify the role of ATG14 as a key regulator in the occurrence of LID, we overexpress ATG14 in the striatum of LID rats. Chronic L‐DOPA treatment induced axial, limb, and orolingual (ALO) AIMs, which progressively increased, reaching the plateau on day 11 and fluctuating afterward (Figure 3C; two‐way repeated measures ANOVA with Bonferroni post hoc tests, treatment: *F*_(3.318,46.45)_ = 765.3, *p* < 0.0001; days: *F*_(1,14)_ = 42.24, *p* < 0.0001; days×treatment: *F*_(8,112)_ = 7.098, *p* < 0.0001). Overexpression of ATG14 consistently reduced the ALO AIMs scores across the whole 15‐day period of L‐DOPA treatment (Figure 3C). A subsequent analysis of individual ALO subitem scores further revealed that ATG14 overexpression exerted a positive role on all three ALO subdomains, as depicted in Figure 3D (two‐way repeated measures ANOVA with Bonferroni post hoc tests, treatment: *F*_(4.811,67.36)_ = 359.9, *p* < 0.0001; days: *F*_(1,14)_ = 30.26, *p* < 0.0001; days×treatment: *F*_(8,112)_ = 3.756, *p* = 0.0006), Figure 3E (two‐way repeated measures ANOVA with Bonferroni post hoc tests, treatment: *F*_(5.024,70.34)_ = 464.2, *p* < 0.0001; days: *F*_(1,14)_ = 31.33, *p* < 0.0001; days×treatment: *F*_(8,112)_ = 3.539, *p* = 0.0011), and Figure 3F (two‐way repeated measures ANOVA with Bonferroni post hoc tests, treatment: *F*_(4.733,66.26)_ = 332.2, *p* < 0.0001; days: *F*_(1,14)_ = 56.07, *p* < 0.0001; days×treatment: *F*_(8,112)_ = 4.893, *p* < 0.0001), respectively. Afterwards, we blocked the autophagy pathway with CQ treatment in ATG14‐overexpressed striatum on day 15. As illustrated in Figure 3G (*F*_(2,21)_ = 19.59, *p* < 0.0001), treatment with CQ led to an increase in the reduced ALO AIMs scores induced by ATG14 overexpression. On day 15, ATG14 overexpression significantly reduced ALO AIMs scores from 20 to 100 min post L‐DOPA administration while CQ treatment completely abolished these ameliorative effects across all observed time points (Figure 3H; two‐way repeated measures ANOVA with Bonferroni post hoc tests, times: *F*_(4.632,97.27)_ = 550.2, *p* < 0.0001; treatment: *F*_(2,21)_ = 19.59, *p* < 0.0001; times×treatment: *F*_(14,147)_ = 3.913, *p* < 0.0001). Specifically, the improvement in all three ALO AIMs subitem scores achieved by ATG14 upregulation was reversed after CQ treatment (Figure 3I; *F*_(2,21)_ = 10.84, *p* = 0.0006 for axial AIMs scores; *F*_(2,21)_ = 19.07, *p* < 0.0001 for limbic AIMs scores; *F*_(2,21)_ = 10.82, *p* = 0.0006 for orolingual AIMs scores). In addition, the outcomes of cylinder tests indicated that L‐DOPA enhanced forelimb motor function of PD rats, and ATG14 intervention did not affect the antiparkinsonian effect of L‐DOPA (Figure 3B). These data imply that ATG14 mitigates ALO AIMs scores in LID rats, particularly during the early to mid‐term following L‐DOPA administration, and across all axial, limb and orolingual subdomains.

### 3.4 Striatal ATG14 Overexpression Promoted Autophagic Flux and ΔFosB Degradation in LID Rats

We then investigated the role and mechanism of ATG14 overexpression in autophagic flux impairment, as well as its effect on ΔFosB aggregation in the striatum of LID rats. The virus‐control rats underwent a similar modeling process to the LID rats and were also treated with 6‐OHDA and L‐DOPA; therefore, their lesioned side exhibited molecular alterations similar to those of the LID rats' lesioned side.

**FIGURE 4:**
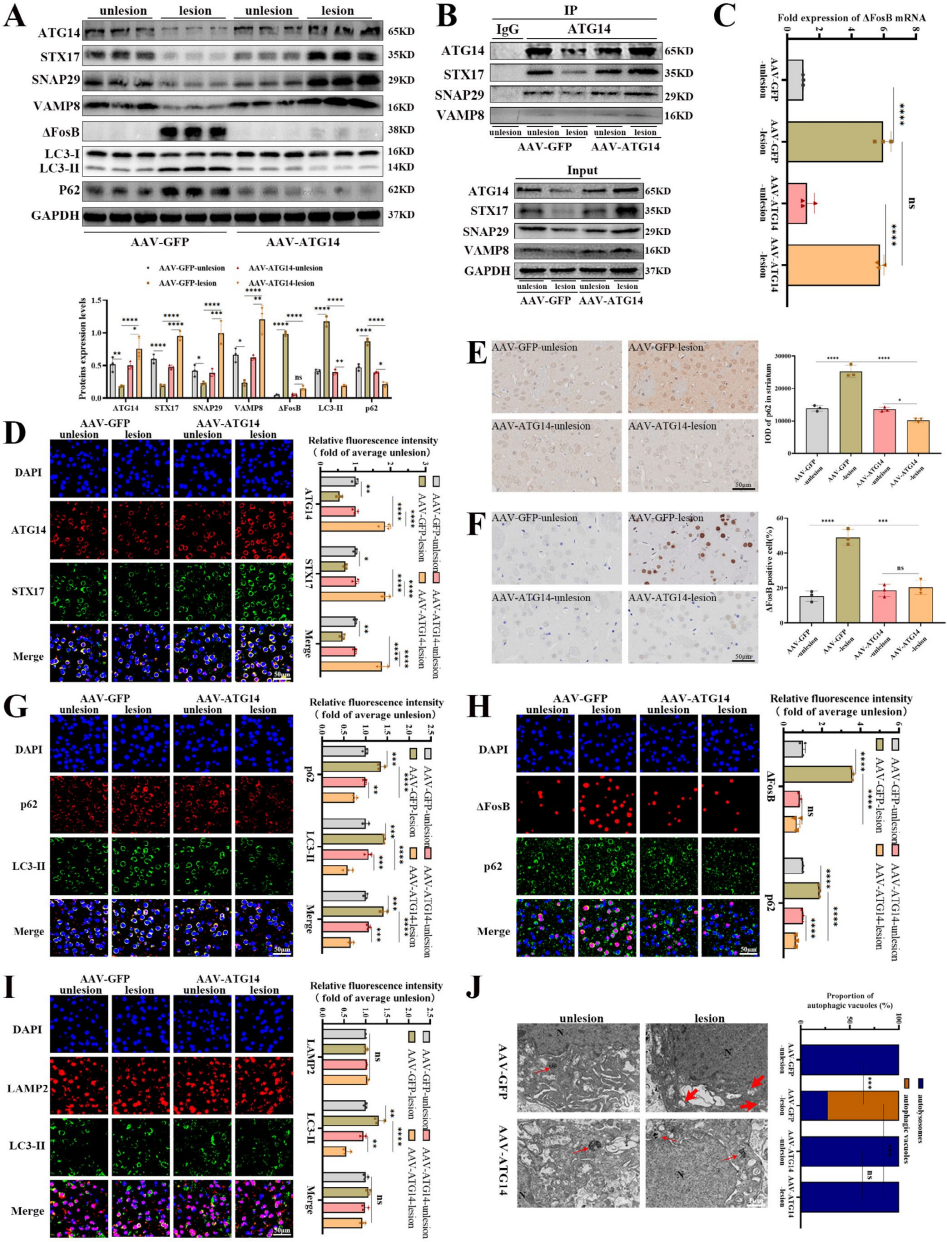
ATG14 overexpression improves SNARE formation, autophagy function and the clearance of ΔFosB in LID rats. (A) The protein levels of ATG14, STX17, SNAP29, VAMP8, LC3, p62, and ΔFosB were detected by western blotting in the striatum of AAV‐GFP and AAV‐ATG14 group rats (*n* = 3). GAPDH as an internal reference. (B) The interaction levels of ATG14 and SNARE complex (namely STX17, SNAP29, and VAMP8) were detected by co‐immunoprecipitation in the striatum of each group (*n* = 1). (C) The ΔFosB mRNA levels in the striatum of each group were detected by real‐time quantitative polymerase chain reaction (*n* = 3). (D, G, H, I) The representative immunofluorescence images of ATG14 and STX17 co‐localization, p62 and LC3‐II co‐localization, LAMP2 and LC3‐II co‐localization as well as p62 and ΔFosB co‐localization in the striatum of each group (*n* = 3; bar = 50 μm). (E, F) The representative immunohistochemical images of p62 and ΔFosB in the striatum of each group (*n* = 3; bar = 50 μm). (J) The representative transmission electron microscopy images of autophagic vacuoles and autolysosomes in each group (*n* = 3; N, nucleus; thin red arrows, autolysosomes; bold red arrows, autophagic vacuoles). (Bars represent the mean, error bars represent the SEM; One‐way ANOVA followed by Tukey multiple‐comparisons tests; *p < 0.05, **p < 0.01, ***p < 0.001, ****p < 0.0001; ns, no significant).

As shown in Figure 4A, the ATG14 protein level (*F*_(3,8)_ = 40.83, *p* < 0.0001) was successfully upregulated by AAV‐ATG14 injection, leading to a consequent rise in STX17 (*F*_(3,8)_ = 104.3, *p* < 0.0001), SNAP29 (*F*_(3,8)_ = 31.12, *p* < 0.0001), and VAMP8 (*F*_(3,8)_ = 32.35, *p* < 0.0001) in the striatum of LID rats. Co‐immunoprecipitation also verified increased interactions between ATG14 and SNARE complex after upregulating ATG14 (Figure 4B and Figure S4). As depicted in Figure 4D, immunofluorescence revealed concurrent increases in ATG14 (*F*_(3,8)_ = 75.96, *p* < 0.0001), STX17 (*F*_(3,8)_ = 112.0, *p* < 0.0001), and their co‐localization (*F*_(3,8)_ = 77.78, *p* < 0.0001) after ATG14 upregulation. As demonstrated in Figure 4I, immunofluorescence showed a significant decrease in LC3‐II (*F*_(3,8)_ = 39.08, *p* < 0.0001), while LAMP2 (*F*_(3,8)_ = 1.561, *p* = 0.2729) and their co‐localization (*F*_(3,8)_ = 2.605, *p* = 0.124) remained unaffected. These data suggest ATG14 overexpression promotes SNARE assembly without influencing lysosome function.

As shown in Figure 4A, ATG14 overexpression decreased p62 (*F*_(3,8)_ = 137.6, *p* < 0.0001) and LC3‐II (*F*_(3,8)_ = 255.6, *p* < 0.0001) protein abundance. Consistently, immunofluorescence confirmed this reduction in p62 (*F*_(3,8)_ = 49.66, *p* < 0.0001) and LC3‐II (*F*_(3,8)_ = 57.93, *p* < 0.0001), as well as their co‐localization (*F*_(3,8)_ = 74.81, *p* < 0.0001) by ATG14 overexpression (Figure 4G). As shown in Figure 4E, immunohistochemistry further supported these findings, showing a decrease in p62 (*F*_(3,8)_ = 96.85, *p* < 0.0001) by ATG14 overexpression. Upon stereotaxic injection of ATG14, a notable decrease in autophagic vacuoles and an increase in autolysosomes were observed (Figure 4J; *F*_(3,8)_ = 24.11, *p* = 0.0002). These data suggest ATG14 overexpression promotes autophagic flux.

ATG14 overexpression reduced ΔFosB protein levels (Figure 4A; *F*_(3,8)_ = 914.0, *p* < 0.0001) and immunoreactive ΔFosB (Figure 4F; *F*_(3,8)_ = 48.57, *p* < 0.0001). Moreover, we discovered that ATG14 overexpression did not significantly alter the transcriptional level of ΔFosB, implying that the effect of ATG14 upregulating ΔFosB was accomplished by promoting protein degradation rather than transcriptional modulation (Figure 4C; *F*_(3,8)_ = 171.8, *p* < 0.0001). Additionally, immunofluorescence demonstrated a consistent decrease in both ΔFosB (Figure 4H; *F*_(3,8)_ = 414.6, *p* < 0.0001) and p62 (Figure 4H; *F*_(3,8)_ = 726.1, *p* < 0.0001), implying that ATG14 overexpression is able to promote autophagy to degrade ΔFosB.

### 3.5 Striatal ATG14 Overexpression Ameliorates Synaptic Disorder in LID Rats

**FIGURE 5:**
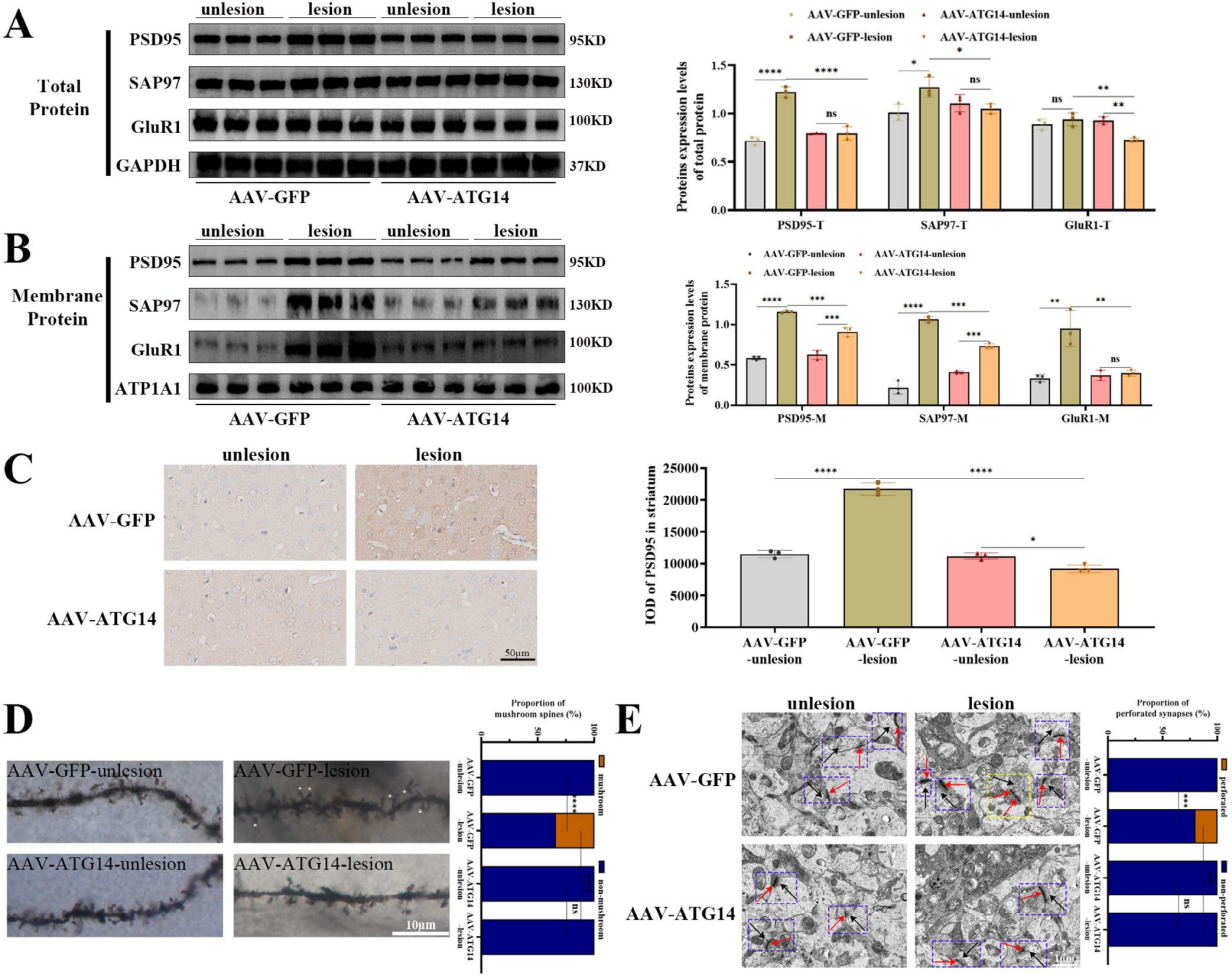
ATG14 overexpression ameliorated maladaptive synaptic structure in LID rats. (A) The total protein levels of PSD95, SAP97, and GluR1 were detected by western blotting in the striatum of AAV‐GFP and AAV‐ATG14 group rats (*n* = 3). GAPDH as an internal reference. (B) The membrane protein levels of PSD95, SAP97, and GluR1 were detected by western blotting in the striatum of each group (*n* = 3). ATP1A1 as an internal reference. (C) The representative immunohistochemical images of PSD95 in the striatum of each group (*n* = 3; bar = 50 μm). (D) The representative Golgi‐stained images of the striatum in each group (*n* = 3; bar = 10 μm; asterisk, mushroom spines). (E) The representative transmission electron microscopy images of perforated synapses and non‐perforated synapses in each group (*n* = 3; bar = 1 μm; black arrows, presynaptic vesicles; red arrows, postsynaptic dense; yellow dashed box, perforated synapses; blue dashed box, non‐perforated synapses). (Bars represent the mean, error bars represent the SEM; One‐way ANOVA followed by Tukey multiple‐comparisons tests; *p < 0.05, **p < 0.01, ***p < 0.001, ****p < 0.0001; ns, no significant).

As shown in Figure 5A, ATG14 overexpression reduced total protein levels of PSD95 (*F*_(3,8)_ = 66.67, *p* < 0.0001), SAP97 (*F*_(3,8)_ = 5.516, *p* = 0.0239), and GluR1 (*F*_(3,8)_ = 12.39, *p* = 0.0022). Membrane protein levels of PSD95 (*F*_(3,8)_ = 119.9, *p* < 0.0001), SAP97 (*F*_(3,8)_ = 179.2, *p* < 0.0001), and GluR1 (*F*_(3,8)_ = 17.90, *p* = 0.0007) were also decreased by ATG14 overexpression (Figure 5B). Immunohistochemistry also revealed a decrease in immunoreactive PSD95 in the striatum of the ATG14 overexpressed LID rats (Figure 5C; *F*_(3,8)_ = 201.4, *p* < 0.0001). Golgi staining exhibited that ATG14 overexpression reduced the ratio of mushroom to non‐mushroom spines in the striatum of these LID rats (Figure 5D; *F*_(3,8)_ = 583.5, *p* < 0.0001). TEM revealed that ATG14 overexpression decreased the ratio of perforated to non‐perforated synapses in the striatum of LID rats (Figure 5E; *F*_(3,8)_ = 31.53, *p* < 0.0001). The above results collectively indicate that ATG14 overexpression is able to attenuate maladaptive synaptic structure in the striatum of the LID rats.

### 3.6 Autophagy Deficits and ΔFosB Accumulation Improved by ATG14 Overexpression Could Be Aggravated by Chloroquine in the Striatum of LID Rats

**FIGURE 6:**
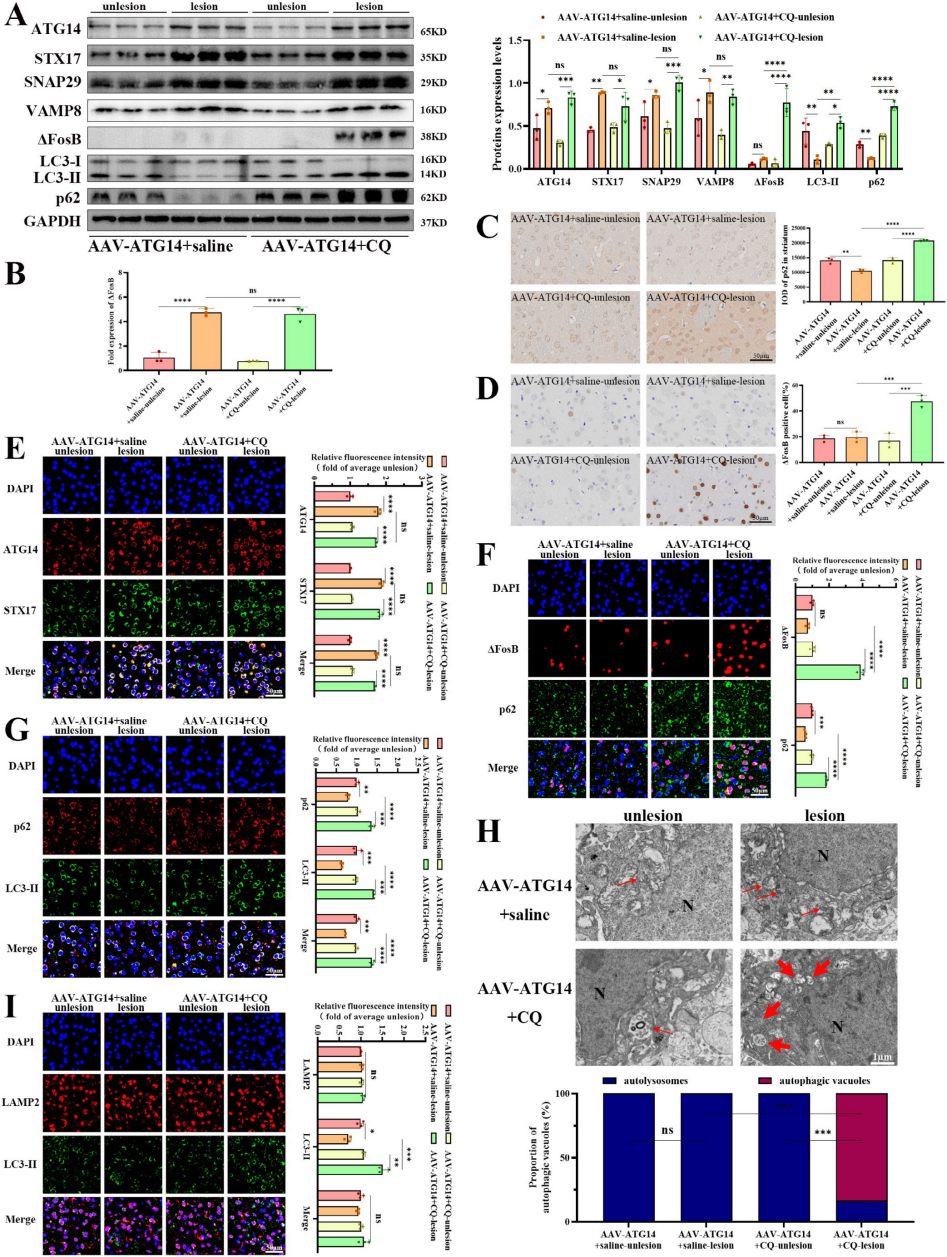
Autophagy deficits and ΔFosB accumulation improved by ATG14 overexpression were aggravated by chloroquine in the striatum of LID rats. (A) The protein levels of ATG14, STX17, SNAP29, VAMP8, LC3, p62, and ΔFosB were detected by western blotting in the striatum of AAV‐ATG14+saline and AAV‐ATG14+CQ group rats (*n* = 3). GAPDH was used as an internal reference. (B) The ΔFosB mRNA levels in the striatum of each group were detected by real‐time quantitative polymerase chain reaction (*n* = 3). (C, D) The representative immunohistochemical images of p62 and ΔFosB in the striatum of each group (*n* = 3; bar = 50 μm). (E, F, G, I) The representative immunofluorescence images of ATG14 and STX17 co‐localization, p62 and LC3‐II co‐localization, LAMP2 and LC3‐II co‐localization as well as p62 and ΔFosB co‐localization in the striatum of each group (*n* = 3; bar = 50 μm). (H) The representative transmission electron microscopy images of autophagic vacuoles and autolysosomes in each group (*n* = 3; N, nucleus; thin red arrows, autolysosomes; bold red arrows, autophagic vacuoles). (Bars represent the mean, error bars represent the SEM; One‐way ANOVA followed by Tukey multiple‐comparisons tests; *p < 0.05, **p < 0.01, ***p < 0.001, ****p < 0.0001; ns, no significant).

After blocking the autophagy flux with CQ, we discovered that protein abundance of ATG14 (*F*_(3,8)_ = 20.41, *p* = 0.4192), STX17 (*F*_(3,8)_ = 16.49, *p* = 0.1972), SNAP29 (F_(3,8)_ = 17.91, *p* = 0.3403), and VAMP8 (*F*_(3,8)_ = 8.786, *p* = 0.9697) remained unaltered (Figure 6A). Immunofluorescence also revealed that ATG14 (*F*_(3,8)_ = 135.1, *p* = 0.5718), STX17 (*F*_(3,8)_ = 352.9, *p* = 0.5720), and their co‐localization (*F*_(3,8)_ = 388.4, *p* = 0.2805) remained unchanged by CQ treatment (Figure 6E). Immunofluorescence showed that LAMP2 levels (*F*_(3,8)_ = 1.342, *p* = 0.3275) were also unaffected by CQ treatment (Figure 6I). These data suggest that CQ does not interfere with either SNARE complex formation or lysosome function.

Unsurprisingly, protein levels of p62 (*F*_(3,8)_ = 179.0, *p* < 0.0001) and LC3‐II (*F*_(3,8)_ = 14.33, *p* = 0.0014) were increased by CQ treatment (Figure 6A). Immunofluorescence also revealed that CQ treatment increased p62 (Figure 6G; *F*_(3,8)_ = 69.27, *p* < 0.0001), LC3‐II (Figure 6G; *F*_(3,8)_ = 84.54, *p* < 0.0001), and their co‐location (Figure 6G; *F*_(3,8)_ = 119.0, *p* < 0.0001). Immunohistochemistry also exhibited elevated p62 (*F*_(3,8)_ = 99.45, *p* < 0.0001) by CQ treatment (Figure 6C). TEM revealed that ATG14 overexpression induced the formation of autolysosomes (thin red arrows), while CQ treatment elicited the formation of autophagic vacuoles (bold red arrows; Figure 6H; *F*_(3,8)_ = 25, *p* = 0.0002). These data imply that CQ treatment halts autophagic flux promoted by ATG14 overexpression.

CQ treatment elevated protein levels of ΔFosB (Figure 6A; *F*_(3,8)_ = 48.76, *p* < 0.0001). Consistent with the western blotting, immunohistochemistry revealed a considerably higher proportion of ΔFosB positive cells (Figure 6D; *F*_(2,6)_ = 34.82, *p* < 0.0001). Moreover, as shown in Figure 6F, immunofluorescence demonstrated a concomitant increase in both ΔFosB (*F*_(2,6)_ = 327.2, *p* < 0.0001) and p62 (*F*_(2,6)_ = 213.7, *p* < 0.0001). Moreover, we discovered that CQ treatment had no obvious effect on the transcriptional level of ΔFosB, implying that autophagic modulation influences ΔFosB primarily through inhibiting protein degradation, rather than transcriptional expression (Figure 6B; *F*_(3,7)_ = 173.5, *p* < 0.0001). These data suggest that CQ treatment reverses ATG14‐mediated autophagic augment, thereby leading to ΔFosB accumulation.

### 3.7 Synaptic Disorder Improved by ATG14 Overexpression Could Be Aggravated by Chloroquine in the Striatum of LID Rats

**FIGURE 7:**
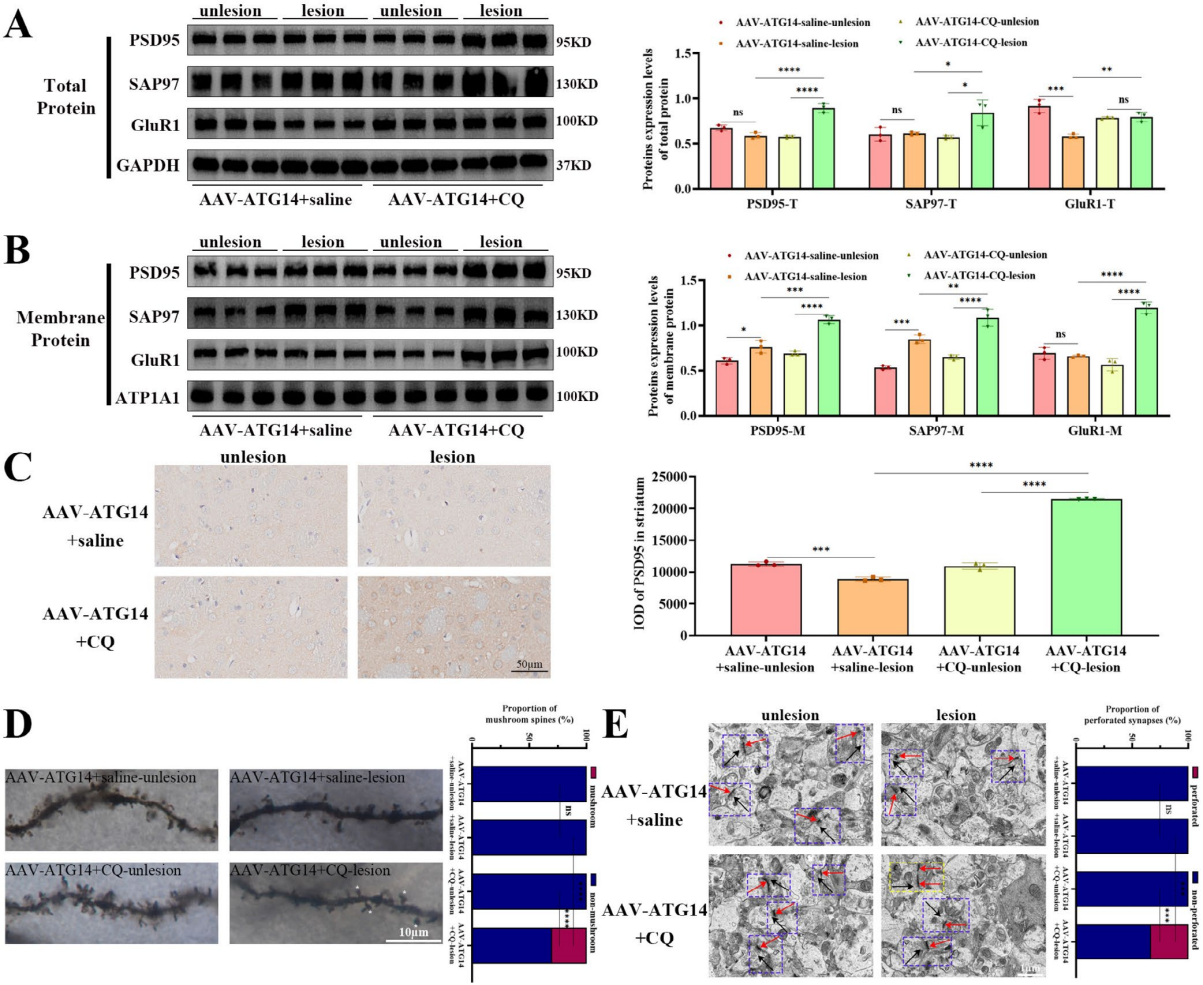
Synaptic disorder improved by ATG14 overexpression was aggravated by CQ in the striatum of LID rats. (A) The total protein levels of PSD95, SAP97, and GluR1 were detected by western blotting in the striatum of AAV‐ATG14+saline and AAV‐ATG14+CQ group rats (*n* = 3). GAPDH as an internal reference. (B) The membrane protein levels of PSD95, SAP97, and GluR1 were detected by western blotting in the striatum of each group (*n* = 3). ATP1A1 as an internal reference. (C) The representative immunohistochemical images of PSD95 in the striatum of each group (*n* = 3; bar = 50 μm). (D) The representative Golgi‐stained images of the striatum in each group (*n* = 3; bar = 10 μm; asterisk, mushroom spines). (E) The representative transmission electron microscopy images of perforated synapses and non‐perforated synapses in each group (*n* = 3; bar = 1 μm; black arrows, presynaptic vesicles; red arrows, postsynaptic dense; yellow dashed box, perforated synapses; blue dashed box, non‐perforated synapses). (Bars represent the mean, error bars represent the SEM; One‐way ANOVA followed by Tukey multiple‐comparisons tests; *p < 0.05, **p < 0.01, ***p < 0.001, ****p < 0.0001; ns, no significant).

As shown in Figure 7A, total protein levels of PSD95 (*F*_(3,8)_ = 49.46, *p* < 0.0001), SAP97 (*F*_(3,8)_ = 6.811, *p* = 0.0136), and GluR1 (*F*_(3,8)_ = 26.18, *p* = 0.0002) were increased by CQ treatment. As shown in Figure 7B, CQ treatment also increased membrane protein levels of PSD95 (*F*_(3,8)_ = 55.06, *p* < 0.0001), SAP97 (*F*_(3,8)_ = 57.74, *p* < 0.0001), and GluR1 (*F*_(3,8)_ = 71.93, *p* < 0.0001). Immunohistochemistry also revealed higher immunoreactive PSD95 by CQ treatment (Figure 7C; *F*_(3,8)_ = 819.5, *p* < 0.0001). Golgi staining revealed that the reduction in mushroom spines induced by ATG14 overexpression was reversed following CQ treatment (Figure 7D; *F*_(3,8)_ = 361, *p* < 0.0001). TEM showed that ATG14 overexpression‐induced reduction of perforated synapses was reversed after CQ treatment (Figure 7E; *F*_(3,8)_ = 31.53, *p* < 0.0001). The above results show that ATG14 overexpression improves synaptic deficits. Conversely, CQ treatment reverses this beneficial effect.

## 4 Discussion

In this study, we found that chronic L‐DOPA treatment caused accumulation of ΔFosB and ATG14‐related autophagy deficiency in the striatum of parkinsonian rats. AAV‐mediated ATG14 overexpression was able to improve autophagic flux, reduce ΔFosB deposition, and thus attenuate the severity of LID by promoting autophagosome‐lysosome fusion. Moreover, chloroquine abolished improvement of ATG14 upregulation on autophagy dysfunction and ΔFosB accumulation. Taken together, targeted activation of the ATG14‐mediated autophagy pathway can digest excessive ΔFosB, which provides a novel approach to treat LID. We showcase the above mechanism in the graphical abstract.

Although the pathogenesis of LID remains unclear, recent research suggests that autophagy is linked to the development of LID (Feyder et al. 2021). Feyder et al. attested that long‐term L‐DOPA regime led to increased p62 in the striatum, more specifically in dSPNs (Feyder et al. 2021). This pilot study suggests a causative link between autophagy deficiency and LID. In line with the above results, our prior study also reported that p62 accumulation mainly occurred in ΔFosB‐labeled cells in the striatum of dyskinetic rats (Liu et al. 2024), while ΔFosB accumulation was predominantly observed in dSPNs (Darmopil et al. 2009). AMPK signaling pathway works through multiple mechanism to regulate autophagy, and activated AMPK could promote autophagy (Wang et al. 2022). We further demonstrated that activation of the AMPK‐mediated autophagy pathway can facilitate ΔFosB degradation and attenuate dyskinetic symptoms, whereas chloroquine counteracted the above improvements (Liu et al. 2024). Chloroquine, being a weak base, hinders lysosomal acidification, thus impeding autophagy by interrupting the fusion of autophagosomes with lysosomes (Maycotte et al. 2012). Combined with observations in TEM, we infer that a disorder of autophagosome‐lysosome fusion occurs in the striatum of dyskinetic rats. Thus we continue to explore the upstream regulator.

A myriad of signal transduction pathways is intimately interwoven to precisely regulate the autophagosome‐lysosome fusion, in which ATG14, STX17, and LAMP2 are key regulators (Diao et al. 2015). To keep autophagic flow working properly, STX17 specifically binds to mature autophagosomes and then interacts with SNAP29 and lysosome‐reside VAMP8 to constitute the SNARE complex (Itakura and Mizushima 2013). ATG14 recruits STX17, stabilizes the STX17‐SNAP29 binary complex, and facilitates fusion with LAMP2‐positive lysosomes, leading to the formation of autolysosomes for autophagic clearance (Diao et al. 2015). In the present study, LAMP2 remained unaltered in LID, suggestive of normal lysosomal function. However, diminished expression of ATG14 and the SNARE complex (namely STX17, SNAP29, and VAMP8) as well as poor interaction between ATG14 and the SNARE complex imply that autophagosome‐lysosome fusion is defective in the striatum of LID rats. Upregulating ATG14 enhanced the expression of SNARE complexes, improved their interaction and co‐localization, and ultimately restored dysfunctional fusion of autophagosomes with lysosomes within the striatum of LID rats. Thus, we have uncovered that ATG14‐mediated autophagosome‐lysosome fusion disorder underlies LID, and that interventions targeting ATG14 may attenuate LID.

Hyperactivation of D1R triggers sustained structural and functional changes in synaptic plasticity. Progressive dopaminergic denervation and chronic dopamine substitution produce dendritic spine pruning and enlarged spine heads in D1R‐SPNs (Nishijima et al. 2018). Expression of postsynaptic protein PSD95 and SAP97, as well as synaptic insertion of AMPARs, contributes to spine enlargement (Nishijima et al. 2018; Rumbaugh et al. 2003; El‐Husseini et al. 2000). Consistent with previous studies (Zhang et al. 2021; Nash et al. 2005; Ba et al. 2006), we observed augmented expression of PSD95 and SAP97 in both total striatum and striatal plasma membrane, while GluR1 was elevated only in the striatal plasma membrane of LID rats. In epileptic rats, LC3‐II directly interacted with PSD95, targeting it for autophagic degradation (Li et al. 2024). However, in models of spinal cord injury, the opposite phenomenon was observed: autophagy activation significantly elevated LC3‐II levels, which were accompanied by a concomitant upregulation of PSD95 expression levels (Wang et al. 2025). This finding suggests that autophagy, besides directly degrading PSD95, may enhance its expression through other mechanisms. In the LID model, we similarly observed significant accumulation of LC3‐II concomitant with an elevation in PSD95 protein levels. We hypothesize that in LID, autophagy may play a role in upregulating PSD95 through indirect regulatory mechanisms. As for SAP97, its relationship with autophagy remains unexplored. Although research revealed that augmented autophagy could degrade intracellular total GluR1 protein (Shehata et al. 2018, 2012), our study failed to detect changes in the overall protein expression of GluR1 by autophagy modulation in LID. In the mouse hippocampus, p62 directly interacted with GluR1, functioning as a scaffold to deliver GluR1 into the postsynaptic membrane (Jiang et al. 2009). Therefore, during the onset of LID, the rise in p62 due to autophagy flux inhibition might directly influence the membrane localization of GluR1.

ΔFosB belongs to the early immediate gene family, and its accumulation has been established to be a dyskinogenic key player (Zamanian et al. 2025). The induction of ΔFosB through the sensitization of dopamine D1 receptors led to subsequent activation of the phosphorylation signaling cascade, which further augmented ΔFosB stability and activity (Zamanian et al. 2025). The absence of a C‐terminal domain renders ΔFosB resistant to ubiquitin‐proteasome degradation (Zamanian et al. 2025). Our earlier study reported that AMPK‐mediated autophagy activation promoted ΔFosB clearance. In this study, with modulation of ATG14, we further revealed the key autophagic target in ΔFosB degradation. Our previous study showed that during LID occurrence, unstable spiking of SPNs and high γ oscillations in the primary motor cortex as well as their synchronization were enhanced, suggestive of maladaptive synaptic changes from an electrophysiological perspective (Zheng et al. 2020). Overexpression of ΔFosB also increased the unstable spiking of SPNs in LID (Cao et al. 2010; Beck et al. 2019). Such neuronal electrical properties hint that ΔFosB might regulate synaptic maladaptation in LID. Therefore, we speculate that amelioration of synaptic maladaptation by ATG14 overexpression could potentially occur through its role in ΔFosB degradation. Although ΔFosB directly binds to GluR2 promoter to induce its transcription in the nucleus accumbens (Nestler 2008), no evidence supports the transcriptional regulation of GluR1, PSD95, and SAP97 by ΔFosB. So further research on how ΔFosB regulates synaptic maladaptation in LID is needed.

This study bears certain limitations. Primarily, our focus was solely on the effects of autophagy regulation on ΔFosB accumulation, while neglecting to explore how ΔFosB might influence the autophagic pathway. This study only elucidates the potential regulatory mechanism of cytoplasmic ΔFosB, and preliminary evidence (unpublished data) suggests that upstream transcription factors upregulate the expression of nuclear ΔFosB, which awaits further validation in subsequent research. It remains to be further elucidated in which subtype of dSPNs the autophagy deficits occur. The contribution of lysosomal dysfunction to autophagic abnormalities in LID also remains to be elucidated. Additionally, it is still unclear whether autophagy dysfunction directly breaks down synaptic proteins. Furthermore, how ΔFosB regulates synaptic changes also needs to be disclosed. Lastly, we preliminarily verified maladaptive synaptic plasticity based on synaptic‐related proteins and synaptic ultrastructure. Corresponding neuroelectrophysiological alterations await further exploration in the future.

## Author Contributions

Conceptualization: Yi Wu and Ke Liu; methodology: Yi Wu, Ke Liu, Zhaoyuan Zhang, and Zhuoran Ma; software: Zhicheng Tang; validation: An Chang; formal analysis: Yi Wu and Haoxuan Ouyang; writing—original draft preparation: Yi Wu; writing—review and editing: Yan Xu and Xuebing Cao; visualization: Heng Zhai; funding acquisition: Yan Xu and Xuebing Cao.

## Funding

This work was supported by the Beijing Medical Award Foundation (Neuroscience Innovation and Development Research Project, Grant No. YXJL‐2022‐0351‐0412).

## Ethics Statement

The animal study was reviewed and approved by the Experimental Animal Management Committee of Tongji Medical College of Huazhong University of Science and Technology (No. 3865).

## Consent

The authors have nothing to report.

## Conflicts of Interest

The authors declare no conflicts of interest.

## Supporting information


**Table S1:** Antibodies used in this study.
**Figure S1:** The original bands of western blotting in Figures 1, 2, 4, and 5.
**Figure S2:** The original bands of western blotting in Figures 6 and 7.
**Figure S3:** Following PD model establishment, total depletion of dopaminergic neurons in the ipsilateral (right) striatum. (A) The representative immunohistochemical images of TH in the striatum of Sham, PD, and LID group rats (*n* = 3; bar = 50 μm). (B) Quantitative analysis of immunohistochemistry. (Bars represent the mean, error bars represent the SEM; One‐way ANOVA followed by Tukey multiple‐comparisons tests; *p < 0.05, **p < 0.01, ***p < 0.001, ****p < 0.0001; ns, no significant).
**Figure S4:** Normalization of the Co‐IP data to the input. (A–D) ATG14, STX17, SNAP29, and VAMP8 Co‐IP/input of Sham, PD, and LID group rats (*n* = 1). (E–H) ATG14, STX17, SNAP29, and VAMP8 Co‐IP/input in the striatum of AAV‐GFP and AAV‐ATG14 group rats (*n* = 1).

## Data Availability

The data that support the findings of this study are available from the corresponding author upon reasonable request.
