# Supplementary material for: ATG14‐Mediated SNARE Complex Activation Promotes ΔFosB Degradation to Ameliorate Levodopa‐Induced Dyskinesia
Source: J Neurochem. 2026 Apr 9;170(4):e70431. doi: 10.1111/jnc.70431 (PMC13063209; doi:10.1111/jnc.70431)
Supplement: Supplementary file 1 — Table S1: Antibodies used in this study. Figure S1: The original bands of western blotting in Figures 1, 2, 4, and 5. Figure S2: The original bands of western blotting in Figures 6 and 7. Figure S3: Following PD model establishment, total depletion of dopaminergic neurons in the ipsilateral (right) striatum. (A) The representative immunohistochemical images of TH in the striatum of Sham, PD, and LID group rats (n = 3; bar = 50 μm). (B) Quantitative analysis of immunohistochemistry. (Bars represent the mean, error bars represent the SEM; One‐way ANOVA followed by Tukey multiple‐comparisons tests; *p < 0.05, **p < 0.01, ***p < 0.001, ****p < 0.0001; ns, no significant). Figure S4: Normalization of the Co‐IP data to the input. (A–D) ATG14, STX17, SNAP29, and VAMP8 Co‐IP/input of Sham, PD, and LID group rats (n = 1). (E–H) ATG14, STX17, SNAP29, and VAMP8 Co‐IP/input in the striatum of AAV‐GFP and AAV‐ATG14 group rats (n = 1). [file JNC-170-e70431-s001.pdf]

ATG14-mediated SNARE complex activation promotes ΔFosB degradation to ameliorate levodopa-induced dyskinesia

Yi Wu[1, #], Ke Liu[2, #], Zhaoyuan Zhang[3], Zhuoran Ma[1], Zhicheng Tang[1], An Chang[1], Haoxuan Ouyang[1], Heng Zhai[1], Xuebing Cao[1,*], Yan Xu[1,*]

# These authors have contributed equally to this work.

* Corresponding authors. E-mail addresses: xuyanwxf@126.com (Y. Xu), caoxuebing@126.com (X. Cao).

1 Department of Neurology, Union Hospital, Tongji Medical College, Huazhong University of Science and Technology, Wuhan, Hubei, China

2 Department of Neurology, Jiangsu Province Hospital of Chinese Medicine, Affiliated Hospital of Nanjing University of Chinese Medicine, No. 155 Hanzhong Road, 210029, Nanjing, China

3 Department of Neurology, the First Medical Center, Chinese PLA General Hospital, Beijing, 100853, China

Supplementary data

Antibodies	Species	Source	RRIDs	Catalogue numbers	Dilution
ATG14	Rabbit	Proteintech	AB_10642701	cat. no. 19491-1-AP	WB, 1:1000<br>IF, 1:500
STX17	Rabbit	Proteintech	AB_2935590	cat. no. 81899-1-RR	WB, 1:10000
SNAP29	Rabbit	Proteintech	AB_2192340	cat. no. 12704-1-AP	WB, 1:3000
VAMP8	Rabbit	ZEN BIO	-	cat. no. R381612	WB, 1:1000
LC3	Rabbit	Proteintech	AB_2137737	cat. no. 14600-1-AP	WB, 1:800
p62	Rabbit	Proteintech	AB_10694431	cat. no. 18420-1-AP	WB, 1:10000<br>IF, 1:800<br>IHC, 1:200
FosB	Rabbit	Cell Signaling Technology	AB_2106903	cat. no. 2251	WB, 1:1000<br>IF, 1:800<br>IHC, 1:200
GAPDH	Mouse	Proteintech	AB_2107436	cat. no. 60004-1-Ig	WB, 1:10000
STX17	Mouse	Santa Cruz Biotechnology	-	cat. no. sc-518187	IF, 1:250
LC3	Mouse	Abmart	AB_3712526	cat. no. M046830	IF, 1:200
LAMP2	Rabbit	Abcam	AB_10971511	cat. no. ab125068	IF, 1:500
p62	Mouse	Novus	-	cat. no.	IF, 1:100

		Biologicals		H00008878-M01	
PSD95	Rabbit	Proteintech	AB_2687961	cat. no. 20665-1-AP	WB, 1:4000<br>IHC, 1:800
SAP97	Rabbit	Abcam	-	cat. no. ab300481	WB, 1:1000
GluR1	Rabbit	Proteintech	AB_2882842	cat. no. 67642-1-Ig	WB, 1:20000

WB, western blotting; IHC, immunohistochemicalstaining; IF, immunofluorescencestaining; RRIDs, research resource identifiers

Supplementary Table 1 Antibodies used in this study.

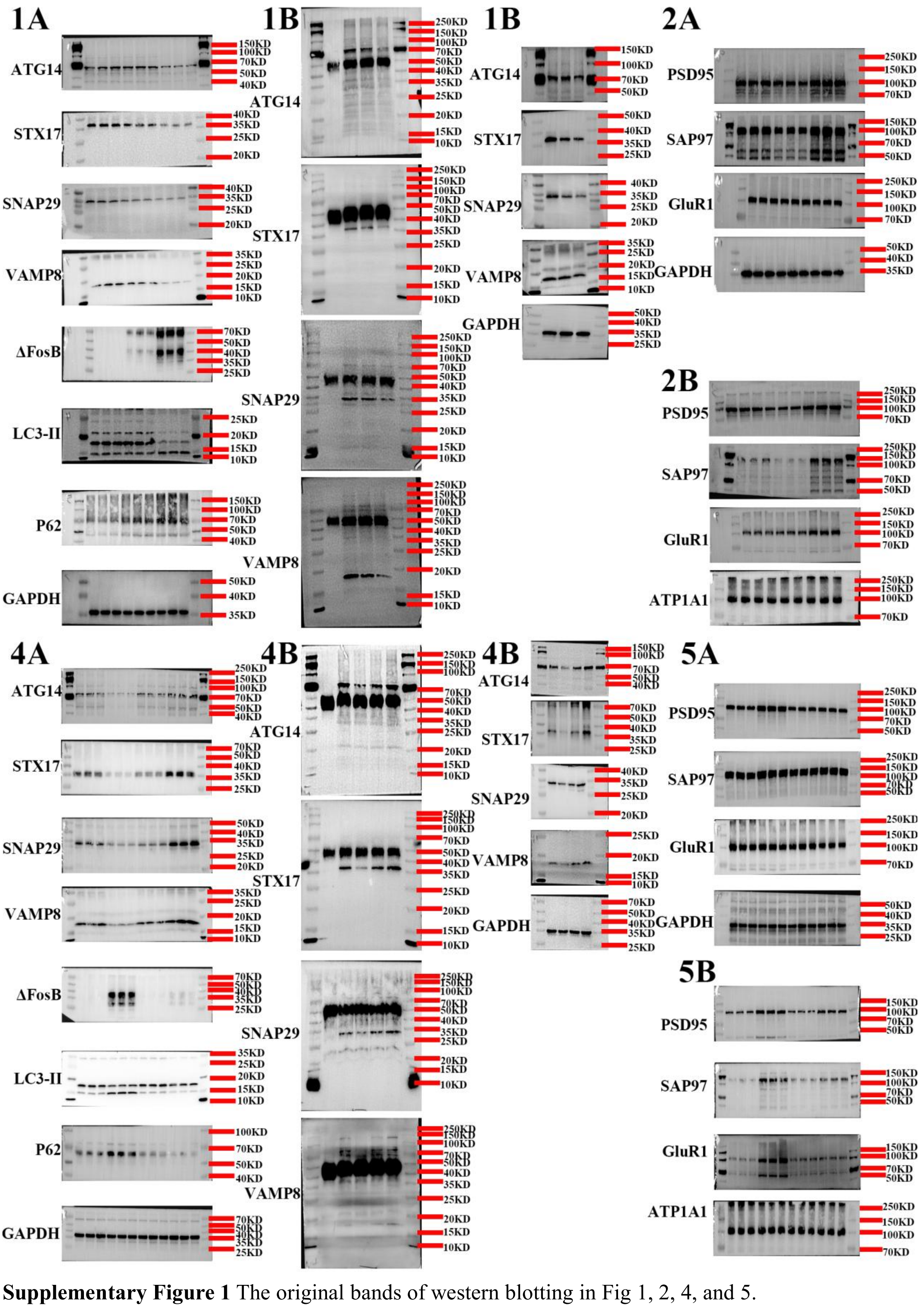

Supplementary Figure 1 The original bands of western blotting in Fig 1, 2, 4, and 5.

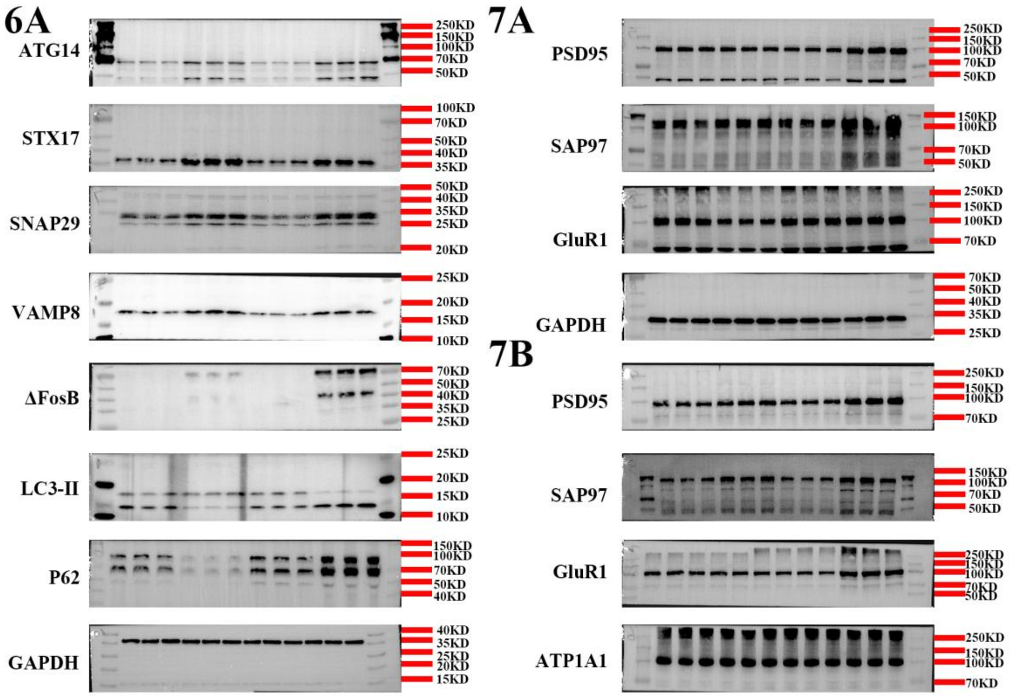

Supplementary Figure 2 The original bands of western blotting in Fig 6 and 7.

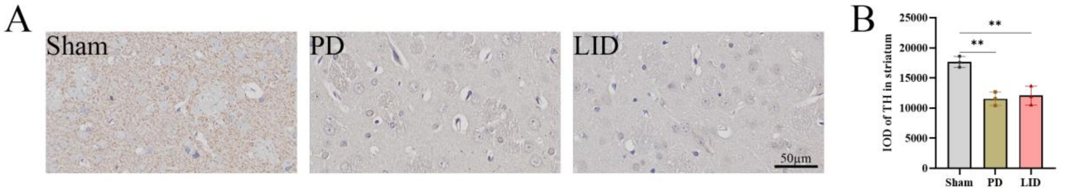

Supplementary Figure 3 Following PD model establishment, total depletion of dopaminergic neurons in the ipsilateral (right) striatum. (A) The representative immunohistochemical images of TH in the striatum of Sham, PD, and LID group rats (n = 3; bar = 50 μm). (B) Quantitative analysis of immunohistochemistry. (Bars represent the mean, error bars represent the SEM; One-way ANOVA followed by Tukey multiple-comparisons tests; *$p < 0.05$, **$p < 0.01$, ***$p < 0.001$, ****$p < 0.0001$; ns, no significant).

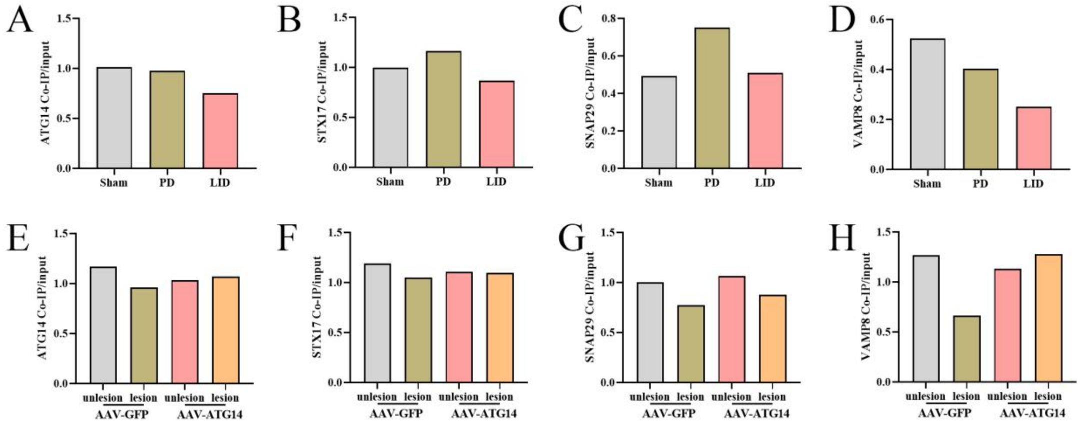

Supplementary Figure 4 normalization of the Co-IP data to the input. (A-D) ATG14, STX17, SNAP29, and VAMP8 Co-IP/input of Sham, PD, and LID group rats (n = 1). (E-H) ATG14, STX17, SNAP29, and VAMP8 Co-IP/input in the striatum of AAV-GFP and AAV-ATG14 group rats (n = 1).
